# Endoplasmic reticulum stress in cardiomyopathies: from the unfolded protein response to therapeutic opportunities

**DOI:** 10.3389/fcvm.2025.1577186

**Published:** 2025-09-08

**Authors:** Yueqing Qiu, Zhenyi Chen, Pingge He, Zhentao Wang

**Affiliations:** ^1^ Basic Medical College of Yunnan University of Chinese Medicine, Kunming, Yunnan, China; ^2^ Second School of Clinical Medicine, Henan University of Chinese Medicine, Zhengzhou, Henan, China; ^3^ Department of Cardiovascular Medicine, Second Affiliated Hospital of Henan University of Chinese Medicine, Zhengzhou, Henan, China

**Keywords:** endoplasmic reticulum stress, unfolded protein response, dilated cardiomyopathy, diabetic cardiomyopathy, arrhythmogenic right ventricular cardiomyopathy, hypertrophic cardiomyopathy

## Abstract

The endoplasmic reticulum (ER), a central organelle responsible for maintaining protein homeostasis, calcium balance, and lipid metabolism, is essential for cardiovascular integrity. Functional disruption—referred to as endoplasmic reticulum stress (ERS)—has been recognized as a major pathogenic driver across diverse cardiovascular disorders. Under pathological conditions such as hypoxia, nutrient deprivation, or infection, sustained ERS activates the unfolded protein response (UPR). While initially adaptive, prolonged or excessive ERS initiates apoptotic cascades, severely impairing cardiomyocyte metabolism, structure, and survival. This review examines the pivotal contribution of dysregulated ERS to the pathogenesis of various cardiomyopathy subtypes, including dilated, diabetic, hypertrophic, and arrhythmogenic right ventricular forms. We outline how ERS fosters maladaptive cardiac remodeling by promoting cardiomyocyte apoptosis and exacerbating oxidative stress, ultimately leading to heart failure. Special attention is given to the complex crosstalk between ERS-related signaling pathways (e.g., PERK, IRE1α, ATF6) and disease progression, with detailed analysis of key regulatory molecules, pathogenic genetic variants, and epigenetic alterations. Integrating recent advances, we highlight the therapeutic potential of targeting ERS pathways as a novel approach to cardiomyopathy treatment, offering a conceptual framework for future translational research and precision medicine strategies.

## 1 Introduction

### 1.1 Epidemiology and clinical challenges of cardiomyopathy

In 1957, Brigden first introduced the term *cardiomyopathy* (non-coronary cardiomyopathy) to describe patients with idiopathic myocardial disease (1). In 1980, a World Health Organization (WHO) special task force, chaired by John Goodwin, proposed the first classification of cardiomyopathies based on structural alterations of the heart and hemodynamic phenotypes. This classification encompassed dilated cardiomyopathy, hypertrophic cardiomyopathy, and restrictive cardiomyopathy (2).

Cardiomyopathy is currently defined as a heterogeneous group of myocardial disorders characterized by impaired mechanical systolic or diastolic function, or abnormalities in the heart's electrophysiological activity. Its pathogenesis is multifactorial, with genetic determinants playing a predominant role, while acquired factors—such as metabolic derangements and toxic insults—also contribute substantially (3, 4). From a genetic perspective, cardiomyopathies can be broadly classified into two categories (4):

(1) Primary cardiomyopathy, in which pathological changes are largely confined to the myocardium. This form may arise directly from genetic abnormalities, as in hypertrophic cardiomyopathy (HCM) (5) and arrhythmogenic right ventricular cardiomyopathy (ARVC) (6), or may be triggered by acquired insults such as systemic injury associated with peripartum cardiomyopathy (7). Importantly, genetic and acquired mechanisms can converge in certain subtypes, including dilated cardiomyopathy (DCM) and restrictive cardiomyopathy (RCM) (5, 8, 9), underscoring that these mechanisms are not mutually exclusive.
(2) Secondary cardiomyopathy, which results from systemic pathological processes originating outside the cardiovascular system, such as metabolic disorders, infections, or drug-induced toxicity. Representative subtypes include diabetic cardiomyopathy (DMCM) (10, 11), sepsis-induced cardiomyopathy (SIC) (12), and doxorubicin-induced cardiomyopathy (DIC) (13).

As cardiomyopathy advances, it often progresses to decompensated heart failure—a critical global public health concern. Heart failure remains a leading cause of cardiovascular mortality and imposes a considerable societal burden owing to its high fatality rate and frequent hospital readmissions (14). Current clinical management primarily involves pharmacological therapies—such as β-adrenergic receptor blockers, angiotensin-converting enzyme inhibitors (ACEIs), angiotensin receptor blockers (ARBs), angiotensin receptor–neprilysin inhibitors (ARNIs), and diuretics—alongside surgical interventions, including left ventricular assist device implantation. The principal therapeutic goals are to alleviate symptoms and delay disease progression (14–16). Although gene-editing technologies, notably CRISPR/Cas9, and gene therapy have shown substantial promise in preclinical studies, their clinical translation is hindered by challenges in delivery efficiency, target specificity, and safety, preventing their adoption as viable treatment modalities (17–19). These limitations underscore the urgent need to further elucidate the molecular mechanisms of cardiomyopathy and identify novel therapeutic targets.

### 1.2 Biological significance of ERS

The ER is a reticular organelle comprising branched tubules and flattened cisternae, responsible for the synthesis, folding, and processing of over one-third of cellular proteins. Proteins destined for the ER, plasma membrane, Golgi apparatus, and lysosomes are co-translationally translocated into the ER lumen via membrane-bound ribosomes, a mechanism that similarly governs the biosynthesis of all secretory proteins. ER-targeted proteins carry N-terminal signal peptides, which are recognized by the signal recognition particle during ribosome binding and subsequently cleaved by signal peptidase during polypeptide elongation (20).

Proper protein folding within the ER lumen relies on a specialized redox environment and a network of enzymatic systems: molecular chaperones prevent aggregation via ATP-dependent binding; glycosyltransferases catalyze N-linked glycosylation; and disulfide isomerases maintain thiol–disulfide bond equilibrium. Sustaining this microenvironment requires energy-intensive active transport, including a Ca^2+^ gradient approximately three orders of magnitude higher than the cytosol and an oxidative potential with a GSSG/GSH ratio > 3:1. Protein maturation involves glycan trimming and remodeling, and only correctly folded molecules, as verified by ER quality control systems, are allowed to assemble into multi-subunit complexes and proceed along downstream secretory pathways (21, 22).

However, stress conditions, including hypoxia, nutrient deprivation, or infection, can compromise the protein-folding capacity of the ER, resulting in the accumulation of unfolded or misfolded proteins. This condition, referred to as ERS, disrupts cardiomyocyte homeostasis and contributes to the pathogenesis of cardiac diseases (23–27). Simultaneously, ERS activates multiple intracellular homeostatic mechanisms, chiefly governed by the dynamic regulation of the UPR. Key processes include IRE1-mediated splicing of XBP1 mRNA to enhance protein-folding capacity, ATF6-driven upregulation of molecular chaperones to remodel the ER microenvironment, and PERK-mediated translational attenuation coupled with induction of pro-apoptotic factors such as CHOP.

The UPR not only directly maintains protein homeostasis (28) but also collaborates with the ER-associated degradation (ERAD) system to clear misfolded proteins (25, 26) and selectively eliminates damaged ER membrane structures via ER-phagy (29).

Nevertheless, in the context of cardiac disease progression, prolonged UPR activation can transition from an adaptive mechanism to a pro-apoptotic signal, promoting cardiomyocyte death or dysfunction through mitochondrial pathways, death receptor signaling, and disruption of calcium homeostasis. Accordingly, this review emphasizes the dual role of the UPR in cardiomyopathies, aiming to clarify how perturbations in its molecular network act as critical drivers of disease onset and progression, thereby offering a conceptual framework for future targeted therapeutic strategies.

## 2 ERS and UPR

ER homeostasis is essential for maintaining cellular physiological function, and its dynamic equilibrium relies on the adaptive regulatory network mediated by the UPR. As the principal response mechanism to ERS, the UPR primarily facilitates the clearance of unfolded proteins and enhances the ER's protein-folding capacity (30). When the UPR successfully mitigates ERS, this negative feedback mechanism can restore ER homeostasis. Conversely, if ERS persists beyond the UPR's compensatory capacity, it activates CHOP-mediated apoptotic signaling cascades or JNK-dependent death receptor pathways, ultimately resulting in irreversible cell death (31–33).

Current evidence demonstrates that the UPR is activated via at least three distinct pathways, each initiated by a specific transmembrane protein on the ER membrane: inositol-requiring enzyme 1 (IRE1), protein kinase RNA-like ER kinase (PERK), and activating transcription factor 6 (ATF6). Under normal physiological conditions, these proteins associate with the chaperone BiP (also known as GRP78), which anchors them to the ER membrane. When misfolded or unfolded proteins accumulate within the ER, these transmembrane proteins dissociate from BiP, thereby activating the IRE1-, PERK-, and ATF6-mediated signaling pathways to alleviate ERS-induced accumulation of unfolded proteins (34, 35).

### 2.1 PERK pathway

PERK is a transmembrane protein with a canonical topology, featuring an N-terminal lumenal domain that directly senses the accumulation of unfolded proteins within the ER lumen and a C-terminal cytoplasmic domain containing a highly conserved serine/threonine kinase motif (36). Under physiological conditions, PERK maintains an inactive conformation by forming a complex with the chaperone immunoglobulin heavy-chain binding protein (BiP). Upon ERS, BiP preferentially binds to exposed hydrophobic regions of unfolded proteins and dissociates from PERK, triggering a conformational rearrangement and initiating PERK autophosphorylation (34).

Activated PERK specifically phosphorylates eukaryotic initiation factor 2α (eIF2α) at Ser51, globally attenuating protein translation while selectively enhancing ribosomal scanning of ATF4 mRNA. The resulting ATF4 protein translocates to the nucleus, where it regulates target gene transcription to participate in three key physiological processes (34): (i) maintenance of amino acid metabolic homeostasis; (ii) regulation of redox balance; and (iii) activation of the ERAD pathway. These coordinated effects significantly enhance the ERS response capacity in cardiomyocytes.

Notably, sustained ATF4 expression initiates a biphasic regulatory mechanism (37). On one hand, it induces GADD34 to form a PP1 phosphatase complex, promoting eIF2α dephosphorylation and restoring protein translation. On the other hand, it upregulates CHOP expression, activating the caspase cascade and ultimately triggering programmed cell death. This finely tuned dynamic balance ensures precise regulation between adaptive responses to ERS and irreversible cardiomyocyte injury.

### 2.2 IRE1 pathway

IRE1α exhibits a topology similar to that of PERK, comprising an N-terminal ER lumenal sensing domain, a single transmembrane helix, and a C-terminal cytoplasmic dual-function catalytic domain. Under ER homeostasis in cardiomyocytes, IRE1α remains inactive through interaction with BiP via its lumenal domain. Upon accumulation of unfolded proteins, BiP dissociates due to affinity switching, triggering IRE1α dimerization, autophosphorylation, and activation of its endoribonuclease activity. Activated IRE1α specifically splices a 26-nt intron from XBP1 mRNA, converting the inactive XBP1u into the transcriptionally active XBP1s isoform. Following this unconventional splicing and re-ligation, the translated XBP1s translocates to the nucleus, where it binds ER stress response elements (ERSE) and activates three major classes of target genes (38): (i) ER chaperone proteins (e.g., PDI, GRP94); (ii) key enzymes involved in lipid biosynthesis; and (iii) components of the ERAD pathway. Collectively, this system enhances the ER's protein-handling capacity.

In addition to its endoribonuclease activity, the cytoplasmic domain of IRE1α possesses serine/threonine kinase function. Through modulation of relevant signaling pathways, this kinase activity cooperatively upregulates the expression of chaperones and folding enzymes, forming a positive feedback loop that mitigates ERS (35, 39). However, under prolonged stress, IRE1α degrades pre-miR-301a, relieving microRNA-mediated repression of GADD45A mRNA, thereby increasing expression of this pro-apoptotic factor. This activates the JNK/ p38 mitogen-activated protein kinase (p38 MAPK) signaling axis and ultimately triggers programmed cell death (40). Such a bimodal regulatory mechanism finely balances adaptive ERS responses and cell fate decisions in cardiomyocytes.

### 2.3 ATF6 pathway

ATF6 exhibits a unique transmembrane topology, with an N-terminal lumenal domain anchored to the ER lumen via glycosylation, enabling real-time monitoring of unfolded proteins, and a C-terminal cytoplasmic domain containing transcriptional regulatory elements. Under conditions of ER homeostasis disruption, ligand-induced conformational changes occur in the lumenal domain of ATF6, triggering its retrotranslocation from the ER membrane to the Golgi apparatus. During this process, ATF6 undergoes sequential proteolytic processing: first, site-1 protease (S1P) cleaves the lumenal regulatory domain, followed by site-2 protease (S2P)-mediated cleavage of the transmembrane segment, ultimately releasing the transcriptionally active N-terminal fragment (ATF6-N) (41, 42).

**Figure 1:**
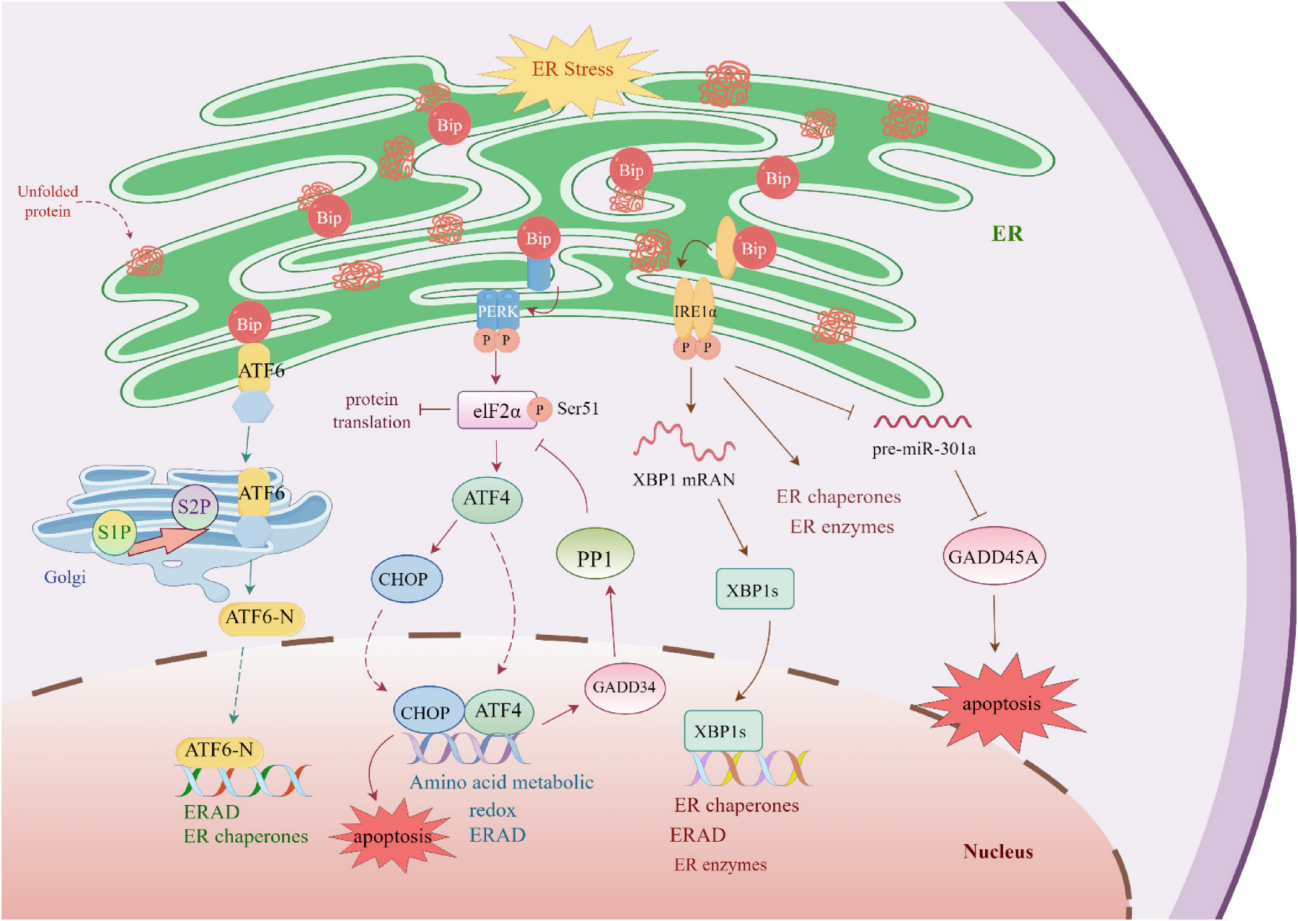
The unfolded protein response in the heart. The accumulation of misfolded and unfolded proteins in the endoplasmic reticulum (ER) triggers the dissociation of Bip from IRE1, PERK, and ATF6, thereby activating three distinct signaling pathways. Upon activation, PERK phosphorylates eukaryotic initiation factor 2α (eIF2α), inhibiting its function and globally suppressing protein synthesis by impairing the activity of 80S ribosomes. However, phosphorylated eIF2α enhances the translation of activating transcription factor 4 (ATF4); ATF4 translocates to the nucleus and regulates stress response pathways, including endoplasmic reticulum-associated degradation (ERAD). Meanwhile, ATF4 induces growth arrest and DNA damage-inducible protein 34 (GADD34), which forms a PP1 phosphatase complex to promote the dephosphorylation of eIF2α (restoring protein translation), and upregulates the transcription of C/EBP homologous protein (CHOP) to promote cardiomyocyte apoptosis. Concurrently, activated IRE1 splices the mRNA of X-box binding protein 1 (XBP1), generating the active XBP1s isoform; this isoform enters the nucleus and upregulates ERAD-related genes. Nevertheless, IRE1 also promotes the degradation of pre-miR-301a, leading to increased expression of GADD45A and subsequent induction of apoptosis. In addition, ATF6 undergoes sequential cleavage by site 1 protease (S1P) and site 2 protease (S2P) in the Golgi apparatus, releasing an active N-terminal fragment. This fragment translocates to the nucleus and acts as a transcription factor to upregulate genes involved in ERAD, thereby enhancing the ER's capacity to restore protein homeostasis.

The proteolytically activated ATF6-N contains a conserved basic leucine zipper (bZIP) domain and rapidly translocates to the nucleus. By binding ER stress response elements (ERSE), ATF6-N directly activates transcription of molecular chaperones (e.g., BiP, GRP94), folding enzymes (e.g., members of the PDI family), and key genes in the ERAD pathway. Moreover, ATF6-N cooperates with other transcription factors, such as XBP1 and ATF4, to form an integrated regulatory network. This multilayered gene expression reprogramming significantly enhances ER protein-folding efficiency and the clearance of misfolded proteins, constituting a crucial cellular defense mechanism against ERS (29, 30, 32). Figure 1 presents the detailed mechanism diagram.

## 3 Primary cardiomyopathies and ERS

### 3.1 Dilated cardiomyopathy

The hallmark pathological feature of DCM is progressive left ventricular dilation accompanied by systolic dysfunction [left ventricular ejection fraction (LVEF) < 40%] (8). Its etiology is highly heterogeneous and can be classified into genetic (e.g., sarcomeric protein gene pathogenic variants) and non-genetic categories (9). Non-genetic pathogenic factors include: (i) hemodynamic overload (e.g., uncontrolled hypertension, valvular regurgitation); (ii) inflammatory or infectious injury (e.g., post-viral myocarditis); and (iii) exogenous toxic exposures (e.g., alcohol, chemotherapeutic agents) (43). Notably, genetic susceptibility can interact synergistically with non-genetic factors, such as through epigenetic regulation (e.g., DNA methylation) or complex gene-environment interactions (44). The disease course exhibits a time-dependent progression, with left ventricular systolic function deteriorating in a stepwise manner from the compensatory to decompensated phase (8, 44), making DCM a leading indication for cardiac transplantation in both adults and children worldwide (45).

Accumulating evidence indicates that ERS-mediated apoptotic pathways play a central role in DCM pathogenesis (46). Bioinformatic analyses of transcriptomic sequencing data reveal 1,068 differentially expressed genes (DEGs) between myocardial tissues of DCM patients and healthy controls. Among these, thymocyte-expressed, positive selection-associated 1 (TESPA1) has been shown to exacerbate ERS-mediated cardiomyocyte apoptosis by dysregulating ER calcium homeostasis and promoting abnormal Ca^2+^ release. Further analyses indicate that other DEGs, including myxovirus resistance protein 1 (MX1), thrombospondin 4 (THBS4), and myosin heavy chain 6 (MYH6), also participate in apoptosis-related signaling networks (47).

#### 3.1.1 Hereditary dilated cardiomyopathy

The role of gene pathogenic variants in the pathogenesis of DCM has been extensively validated. In 2024, a research team from Bangkok investigated a Middle Eastern family affected by hereditary DCM and found that all four affected individuals carried homozygous pathogenic variants in the Bcl-2-associated athanogene 5 (BAG5) gene. Heterozygous carriers exhibited only minor electrocardiographic abnormalities, with no significant echocardiographic changes. Further studies demonstrated that although BAG5 homozygous knockout (BAG5^−/−^) mice did not spontaneously develop a DCM phenotype, under tunicamycin (TN)-induced ERS conditions, BAG5^−/−^ mice showed a marked decrease in LVEF and left ventricular fractional shortening (LVFS) compared with BAG5^+/−^ and BAG5^+/+^ mice. Moreover, myocardial tissue from BAG5^−/−^ mice exhibited upregulated expression of ERS markers (Bip, CHOP) accompanied by a significant increase in cardiomyocyte apoptosis (48). These findings suggest that BAG5 deficiency may facilitate the onset and progression of DCM by exacerbating ERS.

Studies of the Sec1 family domain-containing 1 (*Scfd1*) gene further underscore the critical role of ERS in DCM. *Scfd1* is ubiquitously expressed in zebrafish, including cardiac tissue, and is essential for cartilage formation and fin regeneration. *Scfd1*-deficient zebrafish embryos exhibited pericardial edema and abnormal cardiac morphology (49). Functional disruption of *Scfd1* through knockdown or mutagenesis demonstrated that complete loss of this gene induces severe cardiomyopathic phenotypes, including impaired myocardial contractility, disrupted sarcomere integrity, activation of ERS pathways, and elevated cardiomyocyte apoptosis. These findings suggest that *Scfd1* plays an essential role in cardiac development and myocardial function maintenance by modulating ERS pathways (50).

The *LMNA* gene, located on human chromosome 1 (1q22), encodes nuclear lamins A and C, essential structural components of the nuclear lamina. Through alternative splicing, *LMNA* yields two major isoforms—lamin A and lamin C—critical for maintaining nuclear envelope stability, chromatin organization, gene expression regulation, and cellular mechanotransduction (51).

Pathogenic variants in *LMNA*, such as the nonsense variant R321X, represent a significant genetic etiology of DCM. A clinical and experimental study of an Italian cohort demonstrated that carriers of the R321X variant expressed truncated lamin proteins in both ventricular chambers. These aberrant proteins accumulated pathologically within the endoplasmic reticulum, activating ERS as evidenced by abnormal phosphorylation of the PERK signaling pathway. Mechanistic analyses revealed disrupted ER Ca^2+^ uptake and leakage, substantially perturbing cardiomyocyte Ca^2+^ homeostasis and ultimately triggering apoptosis (52). Therefore, these findings elucidate key mechanisms underlying *LMNA* variant-associated DCM.

#### 3.1.2 Non-hereditary dilated cardiomyopathy

MicroRNAs (miRNAs) play crucial regulatory roles in the pathogenesis of DCM, with miR-16-5p being particularly prominent. As a core member of the miR-16 family, miR-16-5p has garnered extensive attention in cardiovascular research due to its involvement in regulating key biological processes, including cell proliferation, apoptosis, differentiation, and angiogenesis. Dysregulation of its expression is closely associated with cardiomyocyte dysfunction and cardiac remodeling (53, 54).

Studies indicate that miR-16-5p expression levels significantly correlate with the pathological progression of ischemic dilated cardiomyopathy (iDCM). Clinical sample analysis reveals characteristic upregulation of miR-16-5p in the peripheral blood of iDCM patients. *In vitro* experiments demonstrate that artificial overexpression of miR-16-5p in human cardiomyocytes induces ERS, triggering inflammatory responses and enhancing autophagic activity. Pathophysiologically, this miR-16-5p-induced autophagy may represent an adaptive cellular response to protein homeostasis imbalance caused by ERS, manifesting as misfolded protein accumulation or aggregate formation. This mechanism attempts to restore intracellular homeostasis by clearing damaged organelles and macromolecular aggregates prior to apoptosis initiation. However, failure of this compensatory response may accelerate cardiomyocyte apoptosis (55).

Dual-luciferase reporter assays confirm that miR-16-5p directly targets and suppresses ATF6 expression, thereby potentiating ERS-mediated apoptosis *in vitro*. In doxorubicin-induced DCM rat models, decreased expression was observed for long non-coding RNA (lncRNA) AC061961.2, β-catenin, axis inhibition protein 2 (Axin2), cellular myelocytomatosis oncogene (c-Myc), and B-cell lymphoma-2 (Bcl-2), while levels of Bip, CHOP, caspase-3, and Bax were elevated. Mechanistic analyses reveal that lncRNA AC061961.2 attenuates ERS-induced cardiomyocyte apoptosis in DCM models by activating the Wnt/β-catenin signaling pathway (56). This finding elucidates the critical role of ERS in DCM pathogenesis and provides a novel theoretical foundation for non-coding RNA-mediated cardioprotective strategies.

Stromal Interaction Molecule 1 (STIM1), a key Ca^2+^ sensor in the endoplasmic/sarcoplasmic reticulum (ER/SR), is highly expressed in cardiomyocytes and regulates critical cellular metabolic processes. Studies using cardiomyocyte-restricted STIM1 knockout (crSTIM1-KO) mice revealed significant ERS and DCM phenotypes. Specifically, crSTIM1-KO mice developed mitochondrial structural abnormalities and lipid deposition by 12 weeks of age, progressing to pronounced glucose and lipid metabolism dysregulation by 20 weeks (57). Collectively, these findings demonstrate that STIM1 facilitates DCM pathogenesis by modulating ERS and glucolipid metabolic homeostasis.

Ufl1, the Ufm1-specific E3 ligase, exhibits significant enrichment in the ER. This subcellular localization suggests Ufl1 regulates key biological processes maintaining ER homeostasis (58, 59). Ufl1 displays differential expression in cardiac pathology: upregulated in myocardial hypertrophy models but downregulated in failing myocardial tissue from DCM patients. Genetic studies show *Ufl1* knockout mice develop age-dependent cardiomyopathy and heart failure, characterized by cardiac fetal gene reactivation, progressive fibrosis, and systolic dysfunction. Notably, under pressure overload, *Ufl1*-deficient mice exhibit exacerbated hypertrophy, accelerated fibrosis, and worsened cardiac function. Transcriptomic analysis reveals disrupted expression profiles of ER-function-related genes upon Ufl1 ablation, while biochemical assays confirm Ufl1 knockout suppresses the PERK pathway, thereby potentiating ERS-mediated cardiomyocyte apoptosis. Therapeutically, administration of the ER chemical chaperone tauroursodeoxycholic acid (TUDCA) effectively attenuates ERS and improves cardiac function (60). Collectively, these findings demonstrate Ufl1 exerts cardioprotection against pathological remodeling during pressure overload by maintaining ER homeostasis through PERK signaling regulation.

Sulfhydryl oxidase 1 (QSOX1), a pivotal thiol oxidase, shows significant upregulation in acute heart failure (AHF) patients and primarily facilitates protein folding within the ER/SR. Genetic knockout studies reveal that *Qsox1*-deficient mice survive but develop DCM phenotypes. In these knockout models, myocardial tissue exhibits reduced expression of sarco/endoplasmic reticulum Ca^2+^-ATPase 2α (SERCA2α), accompanied by disrupted calcium homeostasis, elevated ERS markers (Bip/CHOP), and persistent UPR activation. Further investigations demonstrate that QSOX1 ablation upregulates endoplasmic oxidoreductin-1α (ERO1-α) and peroxiredoxin-4 (PRDX4). Notably, under isoproterenol (ISO) stimulation, *Qsox1* knockout mice display lower ERO1-α/PRDX4 expression than wild-type counterparts, alongside exacerbated oxidative stress and inflammatory responses (61). These findings indicate QSOX1 maintains cardiac function by regulating ER redox homeostasis and protein quality control.

Parkin, an E3 ubiquitin ligase, regulates multiple cellular physiological processes including ERS (62, 63). Clinical studies reveal concurrent upregulation of Parkin and CHOP in myocardial tissue from DCM patients. Genetic evidence demonstrates that *Parkin* knockout mice exhibit exacerbated pathological ventricular remodeling under stress conditions, accompanied by elevated CHOP expression. *In vitro* experiments further confirm that Parkin depletion in HL-1 cardiomyocytes potentiates CHOP expression and aggravates tunicamycin (TM)-induced cell death. These findings indicate Parkin attenuates persistent ERS-induced apoptosis and mitigates pathological ventricular remodeling by negatively regulating CHOP overexpression (64). This mechanism provides a novel molecular basis for understanding Parkin-mediated cardioprotection.

Autoimmune cardiomyopathy represents a major etiological factor in DCM. Studies detect autoantibodies targeting β1-adrenergic receptors (β1-ARs) in ∼30%–40% of idiopathic DCM patients (65). Among these, anti-β1-ECII antibodies constitute pathogenic autoantibodies specifically recognizing epitopes within the second extracellular loop of β1-AR (β1-ECII) (66). Upon binding β1-ECII, these antibodies disrupt β1-AR physiology and initiate pathological cascades (67). Specifically, anti-β1-ECII antibodies activate aberrant post-receptor signaling [including Ca^2+^/calmodulin-dependent protein kinase II (CaMKII) and p38-MAPK] in cardiomyocytes. This dysregulation induces sustained intracellular Ca^2+^ transient elevation, triggering ERS with upregulated expression of key markers (GRP78/CHOP). Persistent ERS ultimately drives cardiomyocyte apoptosis. Furthermore, these antibodies suppress the cardioprotective Phosphatidylinositol 3-Kinase (PI3K)/Protein Kinase B (Akt)/Signal Transducer and Activator of Transcription 3 (STAT3) pro-survival pathway, exacerbating myocardial injury (68). Thus, preclinical evidence supports targeted p38-MAPK inhibition and PI3K/Akt/STAT3 pathway restoration as potential therapeutic strategies for autoimmune DCM.

#### 3.1.3 Potential therapeutic agents for dilated cardiomyopathy

##### 3.1.3.1 Mulberry leaves

Traditionally used as silkworm feed, mulberry leaves (*Morus alba*) are rich in antioxidant polyphenols—including quercetin, naringenin, and epigallocatechin gallate (69)—which mitigate cardiovascular disease risk (70). Studies demonstrate that 5% mulberry leaf dietary supplementation in rat experimental autoimmune myocarditis (EAM) models induced by cardiac myosin significantly attenuates myocardial fibrosis and improves LVEF and LVFS. This intervention also markedly reduces expression of the ERS marker GRP78. Furthermore, mulberry leaf supplementation suppresses myocardial phosphorylation of endothelin-1 (ET-1) and key MAPK pathway components (Akt, ERK, p38-MAPK, and TGF-β1), as well as vascular endothelial growth factor (VEGF) expression (71).

##### 3.1.3.2 Astaxanthin

Astaxanthin (AST), a natural carotenoid, inhibits ERS (72–74). Cumulative evidence demonstrates its protective effects against diverse cardiovascular disorders (75–77). Alcoholic cardiomyopathy (ACM), a DCM subtype directly mediated by chronic excessive ethanol intake, is clinically classified as acute or chronic, with the latter predominating (78, 79). Epidemiological studies reveal cardiac structural/functional abnormalities in ∼1/3 of chronic alcoholics; moreover, ACM accounts for ∼35% of DCM etiologies (80, 81). Experimental studies confirm AST significantly attenuates ethanol-induced cardiac dysfunction, fibrosis, and pathological remodeling in ACM models. Mechanistic analyses establish that AST-mediated cardioprotection in ethanol-exposed H9c2 cells, primary cardiomyocytes, and ACM mice primarily involves suppression of ERS-associated apoptotic pathways (82), suggesting therapeutic potential for ACM.

##### 3.1.3.3 Quercetin

Inflammation and autoimmune responses contribute to multiple cardiac pathologies and may trigger acute/chronic heart failure. Rat experimental autoimmune myocarditis (EAM) models exhibit pathological features closely resembling human giant cell myocarditis, with recurrent episodes progressing to DCM (83). Quercetin (3,5,7,3′,4′-pentahydroxyflavone), a naturally occurring flavonoid abundant in fruits, herbs, and vegetables, demonstrates broad beneficial bioactivities including anti-inflammatory, antioxidant, and neuroprotective effects (84). Notably, quercetin exerts cardioprotective actions against cardiac inflammation in EAM rats (85).

In EAM models, rats display persistently enhanced ERS accompanied by adverse ventricular remodeling characterized by myocardial fibrosis. Compared with vehicle-treated controls, quercetin-administered EAM rats exhibit reduced myocardial ERS and fibrosis markers [osteopontin (OPN) and TGF-β1], alongside improved cardiac structure and function. Furthermore, quercetin significantly suppresses ET-1 expression and phosphorylation of key MAPK cascade components (Akt, ERK, p38-MAPK) (86), suggesting its cardioprotection involves MAPK signaling modulation.

##### 3.1.3.4 Darbepoetin alfa

Extended pharmacological investigations confirm that darbepoetin alfa treatment in rabbit autoimmune cardiomyopathy models (induced by active immunization with β1-ECII-specific peptides) induces sustained Akt and STAT3 pathway activation, evidenced by significantly increased phosphorylation. Furthermore, this therapy upregulates myocardial erythropoietin receptor (EPOR) expression—diminished in failing hearts—and improves cardiac function in β1-ECII-immunized animals. Functional improvement coincides with reversal of key pathologies: reduced cardiomyocyte apoptosis and cleaved caspase-3 levels; normalized p38-MAPK phosphorylation; attenuated ERS; and restored Bcl-2/Bax ratios. In anti-β1-ECII antibody-treated cultured cardiomyocytes, darbepoetin alfa exerts anti-apoptotic effects via Akt/STAT3 activation. Critically, PI3K inhibitor LY294002 and STAT3-specific peptide inhibitors abrogate these protective effects *in vitro* (87). Thus, darbepoetin alfa ameliorates cardiac dysfunction and slows DCM progression by activating PI3K/Akt/STAT3 signaling and attenuating ERS.

##### 3.1.3.5 Candesartan cilexetil

The renin-angiotensin system (RAS), a key enzymatic cascade regulating cardiovascular homeostasis and disease, critically contributes to DCM pathogenesis (88). Candesartan cilexetil is rapidly hydrolyzed *in vivo* to its active metabolite candesartan—a selective angiotensin II (Ang II) ARB that inhibits RAS to treat DCM-associated heart failure (89).

To elucidate candesartan's cellular mechanisms against DCM, studies in porcine cardiac myosin-induced rat models demonstrate that candesartan treatment significantly reduces cardiomyocyte apoptosis rates (confirmed by TUNEL assay). Concurrently, it downregulates expression of ERS and apoptosis-related proteins (GRP78, TRAF2, IRE-1α, and cleaved caspase-12) while upregulating SERCA2a levels (90). These findings indicate candesartan ameliorates DCM by attenuating ERS, improving calcium homeostasis, and inhibiting apoptosis.

##### 3.1.3.6 Diuretics

Both torsemide and spironolactone treatment attenuate ERS-related marker expression, ameliorate inflammatory states, and reduce myocardial fibrosis biomarkers in porcine cardiac myosin-induced DCM rat models, ultimately improving adverse cardiac remodeling (91).

**Figure 2:**
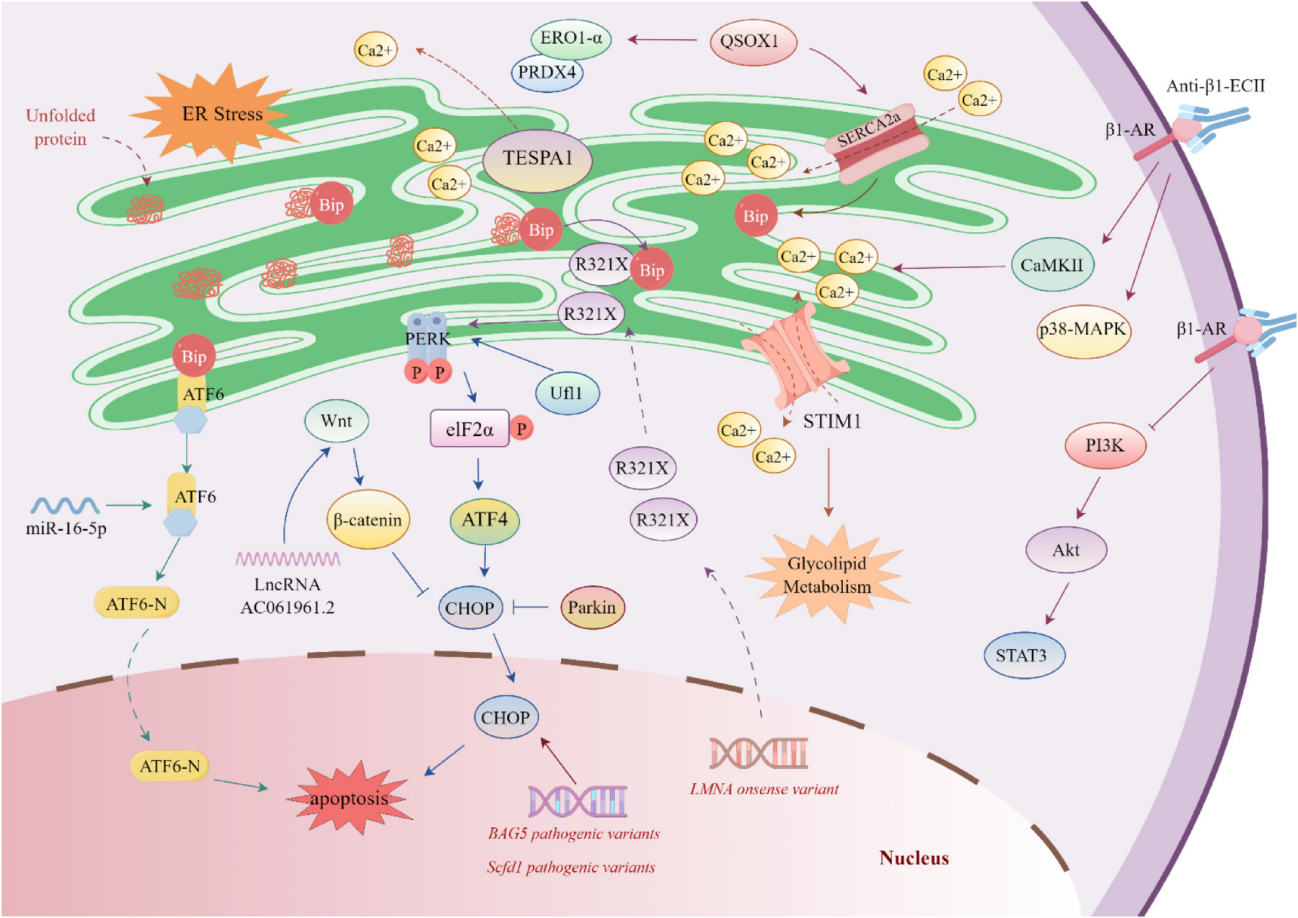
Regulatory mechanisms of endoplasmic Reticulum stress in dilated cardiomyopathy. *BAG5* pathogenic variants increases the expression of immunoglobulin-binding protein (Bip) and C/EBP homologous protein (CHOP), thereby promoting cardiomyocyte apoptosis. The R321X pathogenic variant [a nonsense variant in lamin A (*LMNA*)] leads to the accumulation of abnormal proteins in the endoplasmic reticulum (ER). These abnormal proteins competitively bind to Bip and activate the phosphorylation of RNA-like endoplasmic reticulum kinase (PERK). MicroRNA-16-5p (miR-16-5p) targets and activates activating transcription factor 6 (ATF6). The long non-coding RNA AC061961.2 (lncRNA AC061961.2) can activate the Wnt/β-catenin signaling pathway, inhibit CHOP, and suppress cardiomyocyte apoptosis. Stromal interaction molecule 1 (STIM1) is a calcium sensor in the endoplasmic reticulum/sarcoplasmic reticulum (SR), which regulates calcium homeostasis and glucose-lipid metabolism. Ufm1-specific E3 ligase 1 (Ufl1), enriched in the endoplasmic reticulum, maintains ER homeostasis by regulating the PERK signal. Quinone oxidoreductase 1 (QSOX1) can promote the expression of sarcoplasmic reticulum/endoplasmic reticulum calcium ATPase 2α (SERCA2α) to maintain ER calcium balance, and enhance the expression of ER oxidases—endoplasmic reticulum oxidoreductase 1-α (ERO1-α) and peroxiredoxin 4 (PRDX4). Parkin can inhibit CHOP and alleviate endoplasmic reticulum stress (ERS)-induced cardiomyocyte apoptosis. The anti-β1-ECII antibody specifically recognizes β1-adrenergic receptor (β1-AR), leading to the abnormal activation of post-receptor signaling pathways (including Ca^2+^/calmodulin-dependent protein kinase II (CaMKII) and p38 mitogen-activated protein kinase (p38-MAPK)), and inhibits the PI3K/Akt/STAT3 signaling pathway.

In summary, DCM exhibits etiological heterogeneity encompassing genetic causes (e.g.,*LMNA* pathogenic variants, *BAG5* pathogenic variants) and non-hereditary factors (hemodynamic overload/ inflammation/ exogenous toxicity). ERS-mediated apoptosis constitutes a core pathogenic mechanism: In genetic DCM, BAG5 deficiency, Scfd1 defects, and *LMNA* pathogenic variants (e.g., R321X) exacerbate ERS by disrupting calcium homeostasis and activating the PERK pathway. In non-genetic DCM,miR-16-5p overexpression suppresses ATF6, whereas lncRNA AC061961.2 attenuates ERS damage via Wnt/β-catenin activation.Autoimmune mechanisms (anti-β1-ECII antibodies) induce calcium overload and ERS through p38-MAPK/CaMKII signaling (molecular details in Figure 2). Therapeutic strategies include:Natural agents: Mulberry leaves (inhibit GRP78/MAPK), astaxanthin (antagonizes ERS apoptosis), quercetin (downregulates ET-1/MAPK); Synthetic drugs: Darbepoetin alfa (activates PI3K/Akt/STAT3), candesartan cilexetil (improves calcium homeostasis and inhibits TRAF2/IRE-1α), diuretics (reduce ERS markers) Thus, targeting ERS pathways and their interactomes (calcium homeostasis/autophagy/redox) represents a pivotal therapeutic approach for DCM.

### 3.2 Hypertrophic cardiomyopathy

HCM is a globally prevalent myocardial disorder with an incidence of ∼1:500, primarily driven by sarcomeric gene pathogenic variants. Characteristic pathological features include left ventricular hypertrophy, myocardial fibrosis, hypercontractility, and reduced compliance. Clinical manifestations encompass exercise intolerance, exertional dyspnea, and chest pain (92–95).

Seipin deficiency significantly correlates with HCM progression and heart failure, increasing mortality risk. Compared to wild-type (WT) mice, *Seipin* knockout (SKO) mice subjected to transverse aortic constriction (TAC) exhibit exacerbated left ventricular hypertrophy and diastolic heart failure, accompanied by myocardial inflammatory cell infiltration, collagen deposition, and increased apoptotic bodies. Electron microscopy confirms more pronounced sarcoplasmic reticulum dilation, microtubule disorganization, and mitochondrial impairment in TAC-treated SKO cardiomyocytes. Mechanistically, TAC-induced SKO hearts show upregulated ERS-related genes (e.g., Bip), downregulated SERCA2a and phosphorylated ryanodine receptor (P-RyR) levels, and activated inflammatory/fibrotic pathways. Furthermore, SKO mice exhibit prolonged decay of transient Ca^2+^ currents and sarcoplasmic reticulum Ca^2+^ overload (96).

### 3.3 Arrhythmogenic right ventricular cardiomyopathy

ARVC is a complex genetic disorder with significant clinical implications, strongly associated with ventricular arrhythmias and sudden cardiac death (SCD). Its hallmark pathology features progressive fibrofatty replacement of right ventricular myocardium, accompanied by cardiomyocyte loss leading to wall thinning. Notably, this replacement exhibits distinct spatial heterogeneity, typically initiating subepicardially and extending toward subendocardial regions (97–99).

Studies in ARVC murine models reveal key pathogenic mechanisms: autophagy markers microtubule-associated protein 1 light chain 3 (LC3) and sequestosome 1 (SQSTM1/p62) are significantly overexpressed in cardiomyocytes bordering inflammatory/fibrotic zones, with characteristic autophagic vacuole formation. Early disease stages show upregulated CHOP and XBP1 mRNA in both ventricles, while progressive right ventricular-specific CHOP elevation emerges with disease advancement, concomitant with reduced ryanodine receptor 2 (RyR2) mRNA expression, dilated sarcoplasmic reticulum cisternae, and progressive calcium dysregulation (100).

Desmoglein-2 (*DSG2*) pathogenic variants—the second most frequent genetic cause of ARVC, accounting for ∼10% of cases—cause characteristic pathological changes in human and murine hearts, including cardiomyocyte necrosis, immune infiltration, biventricular fibrofatty replacement, and desmosomal abnormalities. As an ER-synthesized transmembrane protein, *DSG2* undergoes co-translational folding and ER quality control; properly folded proteins are transported to the Golgi for maturation and membrane integration, while misfolded proteins undergo ERS-mediated degradation (101). Mechanistically, DSG2 knock-in mice exhibit myocardial fibrosis and heart failure phenotypes with pronounced PERK/ATF4 pathway activation and TGF-β1 upregulation. Crucially, PERK/ATF4 inhibition attenuates fibrosis (102), confirming cardiomyocyte ATF4/TGF-β1 signaling hyperactivation as a core fibrotic driver and potential therapeutic target.

### 3.4 Restrictive cardiomyopathy

While ERS is implicated in pathological remodeling of dilated and hypertrophic cardiomyopathies, its role in RCM remains poorly characterized. No studies have directly examined ERS activation in human RCM tissues or genetic RCM models. Mechanistically, ERS may exacerbate core RCM pathologies through: (i)Fibrosis acceleration: Chronic ERS upregulates TGF-β1 and phosphorylated p38-MAPK—key drivers of collagen deposition underlying ventricular stiffening (103); (ii)Proteotoxic stress: RCM-associated pathogenic variants cause sarcomeric protein misfolding (104), potentially triggering the UPR; (iii)Ischemia-ERS interplay: Subendocardial ischemia in advanced RCM (103, 104) induces oxidative stress and disrupts ER calcium homeostasis, a mechanism established in ischemic heart disease (36, 105). We propose that ERS potentiates RCM progression *via*: (i) Sustained fibroblast activation promoting irreversible fibrogenesis; (ii) Impaired SERCA2a-mediated calcium reuptake worsening diastolic dysfunction; (iii) CHOP-dependent apoptosis in stiffened myocardium.

## 4 Secondary cardiomyopathies and ERS

### 4.1 Diabetic cardiomyopathy

Diabetes mellitus (DM) constitutes an independent risk factor for heart failure (HF), as established by clinical evidence. DMCM—a diabetes-specific cardiovascular complication—requires strict diagnostic criteria: myocardial structural and functional abnormalities directly attributable to diabetic metabolic dysregulation after excluding coronary artery disease, hypertensive heart disease, valvular pathologies, or other secondary cardiomyopathies. This entity features cardiomyocyte metabolic derangements, myocardial fibrosis, and diastolic/systolic dysfunction, with pathogenesis involving synergistic effects of hyperglycemia, insulin resistance, and dyslipidemia (10, 11, 106, 107). Pathologically, DMCM is characterized by progressive myocardial remodeling and cardiac dysfunction primarily driven by microvascular pathology (diffuse capillary endothelial injury and basement membrane thickening), lipotoxic injury (ectopic lipid deposition-induced cardiomyocyte damage), and oxidative stress/inflammation (chronic oxidative damage synergizing with inflammatory cascades to promote fibrosis and pathological hypertrophy) (106). These interconnected mechanisms amplify through pathological networks to collectively drive DMCM progression.

DMCM exhibits significantly higher incidence in postmenopausal women, suggesting estrogen's potential cardioprotective role against hyperglycemic damage. Persistent ERS is established as a core mechanism in DMCM pathogenesis. Studies demonstrate that activating G protein-coupled estrogen receptor (GPER) ameliorates cardiac structural abnormalities in type 2 diabetes (T2D) rat models through dual molecular mechanisms (108): concomitant downregulation of ERS markers (chaperone Bip, apoptosis-related caspase-12, and pro-apoptotic Bax) and reversal of suppressed SERCA2α and anti-apoptotic Bcl-2 expression. These findings indicate GPER activation attenuates ERS cascades to exert cardioprotection in DMCM, providing a novel therapeutic rationale.

Arrestin domain-containing 4 (ARRDC4), a key arrestin superfamily member, regulates receptor/transporter endocytosis, ubiquitination, and downstream signaling. During hyperglycemia, ARRDC4 upregulation in cardiomyocytes induces glucose transporter 1 (GLUT1) endocytosis via direct binding, limiting glucose uptake (109). This process concurrently activates ERS with elevated CHOP expression. Conversely, ARRDC4-knockout DMCM models exhibit enhanced glucose uptake in cardiac and skeletal muscle, with further observation revealing improved exercise tolerance during diabetes progression (110).

In DMCM, impaired insulin signaling causes abnormal free fatty acid (FFA) accumulation in myocardium, triggering ERS and exacerbating cardiac injury. Interleukin-33 (IL-33) mitigates palmitic acid (PA)-induced ER-associated lipid deposition and cardiomyocyte apoptosis by enhancing autophagy. Insulin-like growth factor binding protein 3 (IGFBP3), a key autophagy regulator, is essential for IL-33-mediated protection since IGFBP3 knockout abolishes these benefits, indicating IL-33 enhances autophagy and ameliorates ERS in an IGFBP3-dependent manner. Promoter deletion analysis reveals IL-33 transcriptionally regulates IGFBP3 by targeting its promoter region (111).

In streptozotocin (STZ)-induced hyperglycemic mice and H9c2 cardiomyocytes, nuclear translocation of spliced X-box binding protein 1 (XBP1s) is significantly attenuated, inversely correlating with its SUMOylation levels. High glucose activates Ras/MEK/ERK signaling, mediating XBP1s phosphorylation at Ser348 and SUMOylation at Lys276. MEK-specific inhibitor U0126 abrogates XBP1s SUMOylation, promotes its nuclear translocation, and ultimately ameliorates DMCM phenotypes (112).

Granulocyte colony-stimulating factor (G-CSF), a key regulator of neutrophil biology, mitigates ERS-induced cardiomyocyte apoptosis in DMCM. By binding specific cell-surface receptors, G-CSF activates intracellular signaling pathways that regulate neutrophil/precursor proliferation, differentiation, functional maintenance, and survival (113, 114). In streptozotocin (STZ)-induced type 1 diabetes (T1D) rat models and high glucose-treated H9c2 cells, G-CSF treatment significantly reduces expression of Bip, caspase-9/12, IRE1α, and CHOP in myocardial tissue and cells (115).

Hydrogen sulfide (H₂S), an endogenous gaseous mediator, critically regulates cardiovascular physiology and pathology (116, 117). Serum H₂S levels are significantly diminished in DMCM patients and animal models (118), with parallel reductions in endogenous H₂S and cystathionine-γ-lyase (CSE) expression in DMCM mice. Exogenous H₂S administration preserves cardiac ultrastructure, attenuating mitochondrial swelling and sarcoplasmic reticulum dilation. Both *in vivo* and *in vitro*, H₂S downregulates ERS-associated proteins—including ATF4, Bip, CHOP, PERK, p-PERK, eIF2α, and p-eIF2α. Mechanistically, H₂S alleviates ERS by targeting muscle RING-finger protein-1 (MuRF1) (119) and mitofusin-2 (Mfn-2) (120), enhancing endoplasmic reticulum-mitochondria interactions and reducing apoptosis.

PI3K is a key intracellular lipid kinase that catalyzes the phosphorylation of the 3-hydroxyl group of the inositol ring in phosphatidylinositol (PI) molecules, generating corresponding 3-phosphorylated phosphatidylinositol derivatives. PI3K exists as a heterodimer composed of a catalytic subunit and a regulatory subunit. The catalytic subunits—p110α, p110β, p110γ, and p110δ—exhibit tissue- and cell type-specific expression patterns and functional specializations, thereby exerting distinct regulatory roles within cellular signaling networks (121–123). PI3K plays a pivotal role in insulin signal transduction; upon insulin binding to its receptor, PI3K is activated, subsequently regulating key cellular responses such as glucose transport and metabolism. Impaired PI3K activation results in defective glucose uptake and metabolism, thereby promoting the onset and progression of DMCM (124). In a T2D mouse model, delivery of constitutively active PI3K (p110α) via recombinant adeno-associated virus rAAV6-caPI3K significantly improved left ventricular function, as evidenced by increased LVEF and LVFS, and downregulated ERS-related markers GRP94 and CHOP, suggesting that PI3K is a potential therapeutic target for DMCM (125).

General control nonderepressible 2 (GCN2), an evolutionarily conserved eIF2α kinase, is activated by accumulated uncharged tRNA during amino acid deprivation. This selectively upregulates amino acid biosynthesis genes to maintain homeostasis (126, 127). GCN2 knockout ameliorates cardiac dysfunction induced by pressure overload (via transverse aortic constriction, TAC) or DOX (128). In STZ-induced T1D and STZ/high-fat diet-induced T2D murine models, GCN2 deficiency significantly attenuates DMCM pathological remodeling—including myocardial hypertrophy, fibrosis, and lipid deposition—concomitant with reduced myocardial p-eIF2α, GRP78, CHOP, ATF4, and Bax levels alongside elevated Bcl-2. These effects were recapitulated in palmitic acid (PA)- or high glucose-treated H9c2 cardiomyocytes (129). Collectively, GCN2 ablation mitigates oxidative stress, inflammation, ERS, and apoptosis in DMCM hearts, ultimately improving cardiac function.

Bromodomain-containing protein 7 (BRD7), a member of the bromodomain protein family, participates in chromatin remodeling and the regulation of gene expression (130). In the hearts of rats with T1D, BRD7 expression is markedly upregulated, whereas its suppression confers protective effects against myocardial injury. In H9c2 cells exposed to high glucose, BRD7 exacerbates ERS-induced apoptosis through activation of the ERK1/2 signaling pathway. Conversely, BRD7 knockdown inhibits the nuclear translocation of XBP1s and reduces CHOP expression levels, further substantiating its critical role in modulating ERS signaling pathways (131).

#### 4.1.1 Potential therapeutic drugs for diabetic cardiomyopathy

Given the critical involvement of ERS in the multifaceted pathogenesis of DMCM, the development of targeted therapeutic strategies is essential. This review highlights pharmacological candidates, including natural compounds and their bioactive constituents, conventional Western medicines, and non-pharmacological interventions such as ketogenic diets and exercise training. These approaches have demonstrated preliminary efficacy in preclinical studies. Tables 1, 2 provide a systematic overview of these candidate agents, detailing their molecular targets and their effects on key pathological processes of DMCM, including myocardial injury, fibrosis, and functional impairment.

**Table 1:** Natural medicines or ingredients for the treatment of diabetic cardiomyopathy.

Drug	Model animal	Effect	Specific manifestation
Daphnetin (132)	STZ-injected diabetic rat model	Inhibits ERS; Inhibits the activation of JNK and MAPK pathways	CHOP↓、GRP78↓、p-PERK/PERK↓、p-JNK/JNK↓、p-p38 MAPK/p38 MAPK↓
D-pinitol (133)	STZ-injected diabetic mouse model; AGEs-induced H9C2 cell model	Inhibits ERS; Inhibits glycophagy; Reduces blood glucose; Alleviates myocardial fibrosis	AGEs↓、GRP78↓、CHOP↓、STBD1↓、GABARAPL1↓、OPTN↑
Polygonatum sibiricum polysaccharide (134)	STZ-injected mouse model after a high-fat diet	Inhibits ERS; Antioxidative stress; Enhances the cGMP-PKG pathway	CHOP↓、GRP78↓、p-PERK/PERK↓、PKG↓、cGMP↑、MDA↓、SOD↑、PDE5↓
Tanshinone IIA (135)	Diabetic mouse model induced by STZ injection after high-fat and high-sugar diet	Inhibits ERS; activates the Sirt1 pathway	GRP78↓、CHOP↓、ATF4↓、ATF6↓、XBP-1s↓、p-eIF2α/eIF2α↓、Sirt1↑
Irisin (136)	H9c2 cell model induced by 33 mM glucose	Inhibits ERS; alleviates oxidative stress	P-PERK/PERK↓、IRE1α↓、GRP78↓
Rutin (137)	High glucose-treated H9C2 cardiomyocyte model	Inhibits ERS; Alleviates cardiomyocyte apoptosis	GRP78↓、IRE1α↓、XBP1↓、ATF6↓、CHOP↓、Cleaved Caspase-3/ Caspase-3↓、Bax/Bcl-2↓
β-carotene (138)	AGE-induced H9c2 cell injury model	Alleviates cardiomyocyte apoptosis; Alleviates oxidative stress; Inhibits ERS; Inhibits AGE-induced autophagy	Bax/Bcl-2↓、Cleaved caspase-3↓、ATF4↓、GRP78↓、CHOP↓、Beclin1↓、LC3Ⅱ/LC3Ⅰ↓、p62↑、p-PI3K/PI3K↑、p-Akt/Akt、p-mTOR/mTOR↑
Astragalus polysaccharide (139)	STZ-injected diabetic rat model; High glucose-induced H9c2 cell model	Inhibits ERS; Alleviates cardiomyocyte apoptosis	ATF6↓、p-IRE1α↓、CHOP↓、p-PERK↓、Caspase-12↓
Matrine (140)	STZ-injected diabetic rat model	Inhibits ERS; Anti-inflammation; Alleviates oxidative stress; Alleviates cardiomyocyte apoptosis	ROS↓、MDA↓、TGF-β↓、PERK↓、IRE1↓、ATF6↓、TNF-α↓、IL-6↓、Caspase-3↓、Caspase-9↓、Bcl-2↑、P53↑
Curcumin (141)	Palmitic acid-treated H9c2 cells	Inhibits ERS; Alleviates cardiomyocyte apoptosis	Caspase-3↓、Bax/Bcl-2↓、CHOP↓、GRP78↓
Ginsenoside Rg1 (142)	STZ-injected diabetic rat model after high-fat and high-sugar diet	Inhibits ERS; Alleviates cardiomyocyte apoptosis	GRP78↓、CHOP↓、Cleaved-Caspase-12↓

**Table 2:** Patent medicines and other therapies for treating diabetic cardiomyopathy.

Drug	Model animal	Effect	Specific manifestation
Eplerenone (143)	Nicotinamide and STZ-induced diabetic rat model	Inhibits ERS; Anti-inflammation	GRP78↓、XBP1↓、IL-1β↓、NLRP3↓
Ranolazine (144)	Nicotinamide and STZ-induced diabetic rat model	Inhibits ERS; Anti-inflammation; Alleviates oxidative stress;	GRP78↓、XBP1↓、SOD↑、GSH↑、MDA↓、IL-1β↓、NLRP3↓
Empagliflozin (145)	STZ-injected diabetic rat model after high-fat diet	Inhibits ERS; Improves cardiac function; Reduces blood glucose	CHOP↓、XBP1↓、ATF4↓、Caspase-12↓、TRAF2↓
Trimetazidine (146)	db/db spontaneous diabetic mouse model	Inhibits ERS; Alleviates cardiomyocyte apoptosis	GLU↓、TC↓、TG↓、LDL-C↓、CK-MB↓、Caspase-12↓
Relaxin-3 (147)	STZ-injected diabetic rat model	Inhibits ERS; Alleviates myocardial fibrosis	IL-17↓、TNF-α↓、GRP78↓、CHOP↓
Ticagrelor (148)	High glucose-incubated H9c2 cell model	Inhibits ERS; Alleviates cardiomyocyte apoptosis; Enhances the migration and tube formation ability of endothelial cells	ROS↓、Bax/Bcl-2↓、GRP78↓、CHOP↓、Ent1↓
Sacubitril/Valsartan (149)	STZ-injected diabetic rat model after high-fat diet	Inhibits ERS; Alleviates cardiomyocyte apoptosis; Anti-inflammation	GRP78↓、PERK↓、eIF2a↓、ATF4↓、CHOP↓、Bax/Bcl-2↓、AGEs↓、NF-*κ*B↓
Zonisamide (150)	STZ-injected diabetic mouse model after high-fat diet; High glucose-induced primary neonatal rat cardiomyocyte injury model	Inhibits ERS; Improves cardiac dysfunction; Inhibits myocardial hypertrophy;	Bax/Bcl-2↓、Cleaved Caspase-3/ Caspase-3↓、GRP78↓、XBP1s↓、ATF6↓、p-PERK↓、ATF4↓、IRE1α↓、CHOP↓、hrd1↑
Spermine (SPM) (151)	STZ-injected diabetic rat model; High glucose-induced cardiac fibroblast model	Inhibits ERS; Inhibits abnormal proliferation and collagen deposition of CFs; Inhibits the Wnt/β-catenin pathway	GRP78↓、GRP94↓、CHOP↓、ATF4↓、Collagen-I↓、Collagen-III↓、MMP-2↓、MMP-9↓、α-SMA↓、Wnt3↓、p-β-catenin/β-catenin↑、Axin2↑
Melatonin (152)	STZ-injected diabetic rat model after high-fat diet	Inhibits ERS; Alleviates cardiomyocyte apoptosis	Caspase-9↓、Caspase-3↓、Caspase-12↓、Bax/Bcl-2↓、CHOP↓、GRP78↓、PERK↓、IRE1α↓、ATF6α↓
N-acetylcysteine (NAC) (153)	Palmitic acid-cultured neonatal rat cardiomyocyte model	Inhibits ERS; Alleviates cardiomyocyte apoptosis; Alleviates oxidative stress	Bax/Bcl-2↓、p-PERK/PERK↓、p-IRE1α/IRE1α↓、p-eIF2α/eIF2α↓、sXBP1↓、GRP78↓、ATF6↓、CHOP↓、Cleaved-Caspase-3↓、ROS↓、NOX2↓、NOX4↓
Erythropoietin (EPO) (154)	STZ-injected diabetic rat model; High glucose-treated neonatal rat cardiomyocytes	Inhibits ERS; Alleviates cardiomyocyte apoptosis	GRP78↓、SERCA2a↑、EPOR↑
Bis(maltolato)oxovanadium(IV) (BMOV) (155)	STZ-injected diabetic rat model after high-sugar diet; High glucose-treated H9C2 cardiomyocyte model	Inhibits ERS; Alleviates cardiomyocyte apoptosis	EIf2a↓、GRP78↓、ATF4↓、ATF6↓、CHOP↓、TRAF2↓、IRE1↓、PERK↓、Caspase-3↓、Caspase-12↓
H2 relaxin/H3 relaxin (156)	High glucose-treated neonatal rat cardiomyocyte model	Inhibits ERS; Alleviates cardiomyocyte apoptosis	Cleaved-Caspase-8↓、Cleaved-Caspase-9↓、 Cleaved-Caspase-12↓、CHOP↓
Hydrogen molecule (157)	STZ-injected diabetic mouse model	Inhibits ERS; Anti-inflammation; Alleviates oxidative stress; Alleviates cardiomyocyte apoptosis	SOD1↑、NOX4↓、CHOP↓、ATF6↓、GRP78↓、p-PERK↓、p-p38/p38↓、p-JNK/JNK↓、NF-κB p65↓、IκBα↑
ZnSO₄ (158)	STZ-injected diabetic rat model after high-fat diet;	Inhibits ERS; Inhibits autophagy	LC3↓、GRP78↓
Irbesartan (159)	STZ-injected diabetic rat model after high-fat diet;	Inhibits ERS; Alleviates cardiomyocyte apoptosis; Inhibits protein kinase D (PKD)	p-PKD↓、GRP78↓、Bax↓、Bcl-2↑、Cleaved-Caspase-3↓
Exercise training (160)	STZ-injected diabetic rat model	Inhibits ERS; Alleviates cardiomyocyte apoptosis	CHOP↓、GRP78↓、Caspase-12↓
Ketogenic diet (161)	STZ-injected diabetic rat model	Inhibits ERS; Anti-inflammation; Alleviates myocardial fibrosis; Alleviates cardiomyocyte apoptosis	CD-36↓、CPT-1β↓、TNF-α↓、IL-1β↓、IL-6↓、PERK↓、eIF2α↓、Bcl-2/BAX↑、Caspase-3↓

**Figure 3:**
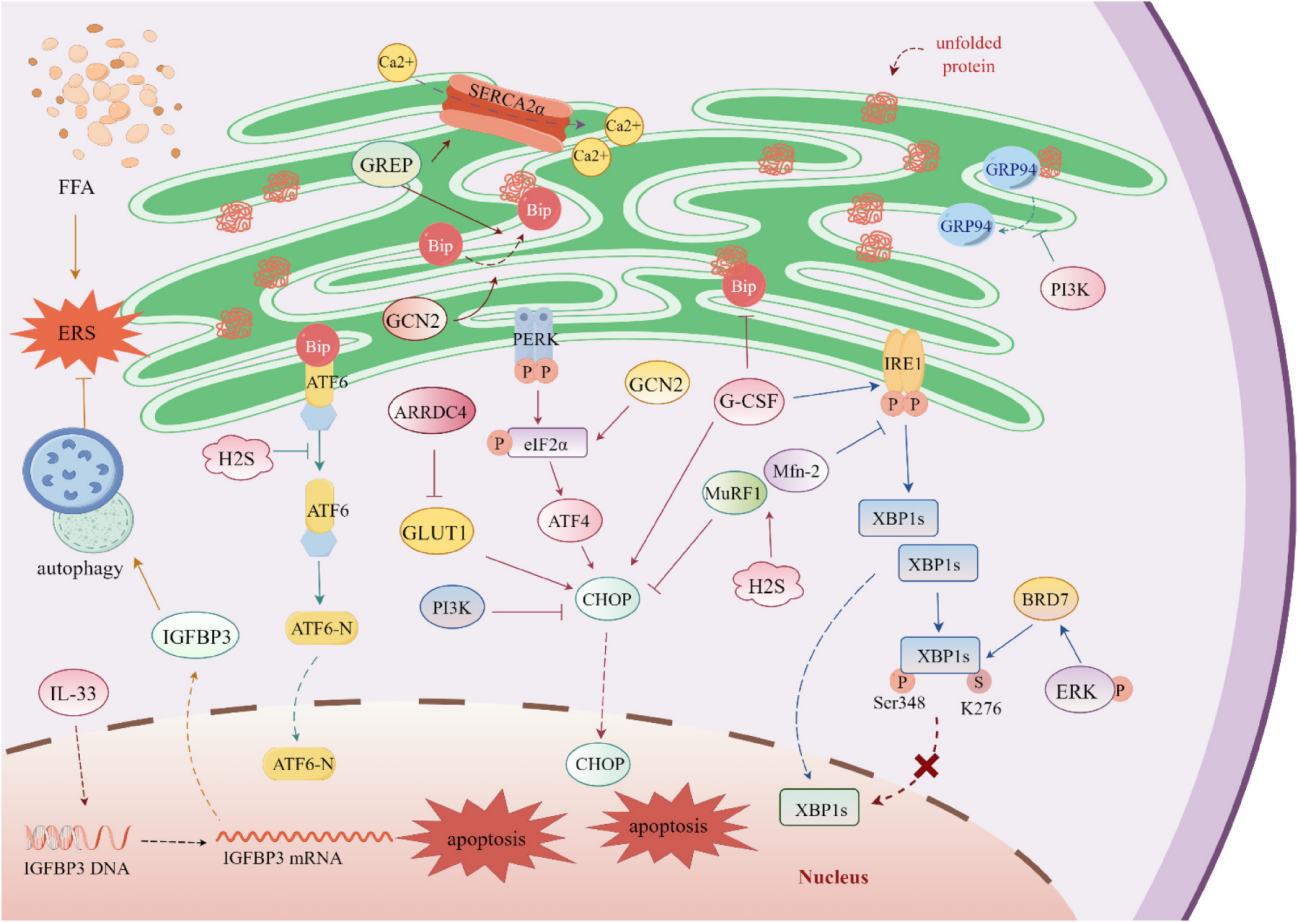
Regulatory mechanisms of endoplasmic Reticulum stress in diabetic cardiomyopathy. The G protein-coupled estrogen receptor (GPER) promotes the binding of immunoglobulin-binding protein (Bip) to misfolded proteins. The expression of sarcoplasmic reticulum calcium ATPase 2α (SERCA2α) is regulated by arrestin domain-containing protein 4 (ARRDC4); upon binding to glucose transporter 1 (GLUT1), ARRDC4 inhibits GLUT1 activity and simultaneously upregulates C/EBP homologous protein (CHOP). Excessive free fatty acid (FFA) accumulation induces endoplasmic reticulum stress (ERS). Interleukin-33 (IL-33) targets the promoter region of insulin-like growth factor-binding protein 3 (IGFBP3), activates its transcription, and enhances autophagy to alleviate ERS. Under high-glucose conditions, X-box binding protein 1 (XBP1s) undergoes SUMOylation and phosphorylation, which inhibits its nuclear translocation. Extracellular signal-regulated kinase (ERK) promotes the expression of bromodomain-containing protein 7 (BRD7); BRD7 inhibits the nuclear translocation of XBP1s without affecting the total expression level of XBP1s. Granulocyte colony-stimulating factor (G-CSF) prevents the dissociation of Bip and activates inositol-requiring enzyme 1 (IRE1) and CHOP. Hydrogen sulfide (H₂S) targets muscle RING-finger protein 1 (MuRF1) and mitofusin 2 (Mfn-2), reducing the expression of multiple ERS-related proteins. Phosphatidylinositol 3-kinase (PI3K) decreases the expression of CHOP and inhibits the dissociation of glucose-regulated protein 94 (GRP94). General control nonderepressible 2 (GCN2), a eukaryotic translation initiation factor 2α (eIF2α) kinase, promotes the expression of multiple ERS-related proteins and enhances cell apoptosis.

In summary, ERS plays a central regulatory role in DMCM. GPER activation antagonizes ERS-induced apoptosis by downregulating Bip/caspase-12/Bax while upregulating SERCA2α/Bcl-2; ARRDC4 activates the CHOP pathway via GLUT1 endocytosis-mediated glucose uptake inhibition; Ras/MEK/ERK signaling inhibits XBP1s nuclear translocation through SUMOylation, which MEK inhibitor U0126 reverses; H₂S targets MuRF1/Mfn-2 to enhance ER-mitochondria interactions, reducing PERK/eIF2α phosphorylation. Novel metabolic interventions include: the IL-33/IGFBP3 axis (IGFBP3-dependent) enhancing lipophagy; GCN2 ablation improving amino acid homeostasis; and PI3K activation (via rAAV6-caPI3K delivery) restoring insulin signaling. Conversely, epigenetic regulator BRD7 exacerbates ERS-apoptosis through ERK1/2 signaling (molecular details in Figure 3).

Therapeutic strategies—natural agents (e.g., daphnetin, tanshinone IIA, astragalus polysaccharides), pharmacologic agents (e.g., empagliflozin, sacubitril/valsartan), and non-pharmacologic interventions (e.g., exercise training)—all attenuate myocardial injury by suppressing core ERS markers (GRP78, CHOP, PERK) via shared anti-inflammatory, antioxidant, and anti-apoptotic mechanisms. Natural agents additionally modulate downstream ERS pathways (e.g., JNK/MAPK, Sirt1, cGMP-PKG), often exhibiting glycemic control and anti-fibrotic effects, while pharmacologic and non-pharmacologic approaches (e.g., ketogenic diet) primarily improve cardiac function and metabolic dysregulation. Future research should delineate synergistic natural/pharmacologic combinations (e.g., natural agents with empagliflozin), elucidate non-pharmacologic regulation of ERS-autophagy networks, expedite clinical translation, and investigate ERS interactions with metabolic reprogramming and immune microenvironments to advance targeted therapies.

### 4.2 Peripartum cardiomyopathy

Peripartum cardiomyopathy (PPCM) is a rare cardiomyopathy clinically characterized by ventricular dilation with systolic dysfunction, most commonly occurring in late pregnancy or the early postpartum period. Although more than 50% of patients can recover systolic function, some may progress to chronic cardiomyopathy, and a small number may even require mechanical support, heart transplantation, or a combination of both (162, 163). Studies have confirmed that a persistent state of ERS activation exists in the myocardial tissue of patients with PPCM. This abnormal ERS signaling may contribute to promoting cardiomyocyte remodeling and metabolic reprogramming through mechanisms such as interfering with myocardial energy metabolism, impairing calcium homeostasis (by regulating the expression of phospholamban (PLN) and SERCA, and disrupting endocytic pathways, thereby facilitating the development and progression of left ventricular dysfunction (164).

### 4.3 Sepsis-induced cardiomyopathy

Sepsis is a life-threatening disease triggered by a dysregulated host response to infection. Sepsis-induced cardiomyopathy (SCM) refers to myocardial dysfunction occurring during sepsis, with the exclusion of other known cardiac diseases (165). SCM is accompanied by intense proinflammatory responses and marked oxidative damage, and is associated with mechanisms such as dysregulated ERS and autophagy (166, 167). Although SCM exhibits a certain degree of reversibility, it significantly increases the risk of death in septic patients. Most cases of sepsis originate from bacterial infections, particularly infections with Gram-negative bacteria (168). Endotoxin (i.e., lipopolysaccharide, LPS), as a fundamental component of the outer membrane of Gram-negative bacteria, can trigger cytokine storms, fatal endotoxemia, and septic shock (169).

The intraperitoneal LPS-injected SCM mouse model demonstrates elevated autophagy markers: increased LC3BII/LC3BI ratio, upregulated Atg7 and Beclin1 expression, and downregulated p62, with concomitant AMPK/ACC pathway activation. ERS markers CHOP, GRP78, and IRE1α are significantly elevated, though eIF2α phosphorylation remains unaffected. Metallothionein (MT) overexpression—a heavy metal scavenger—exerts no intrinsic cardiac effects but attenuates LPS-induced ERS without altering autophagy-related proteins or signaling. *In vitro* studies confirm that inhibiting oxidative stress and ERS protects against LPS-induced myocardial dysfunction (170).

In cecal ligation and puncture (CLP)-induced SCM mice, myocardial ERS proteins show marked alterations: increased p-PERK levels and p-PERK/PERK ratio, alongside downregulated CHOP, ATF6, and GRP78. Pro-apoptotic Bax increases while anti-apoptotic Bcl2 decreases—abnormalities reversed by intraperitoneal zero-valent iron nanoparticles (nanoFe, 20 mg/kg) (171). Furthermore, SCM reduces key mitochondrial biogenesis molecules (SIRT1, PGC-1α, TFAM), UCP2, COXIV, and AMPK/ACC phosphorylation, all rescued by nanoFe treatment (171). These findings indicate nanoFe ameliorates SCM by restoring ERS homeostasis and metabolic pathway function.

### 4.4 Doxorubicin-induced cardiomyopathy

Doxorubicin (DOX), a classic anthracycline chemotherapeutic agent, exhibits well-established anticancer efficacy through decades of clinical application and is widely administered for various malignancies as monotherapy or in combination regimens. Its antitumor mechanism involves targeting topoisomerase IIα and inhibiting DNA/RNA synthesis. However, DOX demonstrates significant cardiotoxicity ranging from arrhythmias to myocarditis and cardiomyopathy. Doxorubicin-induced cardiomyopathy (DIC), classified as non-ischemic cardiomyopathy, is clinically characterized by left ventricular dilation with systolic dysfunction (172–174). Substantial evidence indicates that sustained ERS and calcium dyshomeostasis in DIC trigger cardiomyocyte apoptosis, thereby initiating or exacerbating myocardial injury (175, 176).

In DIC mouse models, myocardial miR-378 expression is significantly downregulated. Mechanistic studies demonstrate that miR-378 attenuates ERS and reduces DOX-induced cardiomyocyte apoptosis by targeting calumenin (177). Sprague-Dawley rat cardiomyocytes treated with DOX exhibit marked ERS activation, characterized by upregulated GRP78, GRP94, and protein disulfide isomerase (PDI) expression, ATF6α cleavage, and increased XBP1 levels. These ERS-related changes are mitigated by IL-10 treatment, which concurrently decreases apoptosis (178).

Under physiological conditions, calmodulin (CaM) localizes along cardiomyocyte Z-lines, forming stable complexes with ryanodine receptor 2 (RYR2) through high co-localization. Following DOX exposure (1 mmol/L, 5 min), CaM dissociates from RYR2. Both dantrolene (DAN) treatment and the RYR2 V3599K variant—which enhances CaM-RYR2 binding affinity—maintain this interaction, reduce calcium leakage, and thereby preserve contractile function. Notably, this protective mechanism operates independently of antioxidant pathways, as evidenced by its insensitivity to N-acetylcysteine (NAC) treatment (179).

A rat model with concurrent myocardial injuries of doxorubicin-induced cardiomyopathy (DIC) and diabetic cardiomyopathy (DMCM) was established via sequential intraperitoneal injection of streptozotocin (STZ) and doxorubicin (DOX). Histopathological evaluation revealed that the degree of cardiomyocyte degeneration in model rats was exacerbated after DOX injection. Following combined intervention with dapagliflozin (DAPA) and trimetazidine (TMZ), the expression levels of GRP78 and CHOP were significantly downregulated, and endoplasmic reticulum stress (ERS) was alleviated. Notably, combined therapy or TMZ monotherapy exerted a more potent effect on alleviating ERS than DAPA monotherapy, confirming the synergistic therapeutic potential of DAPA and TMZ (180). *In vitro* experiments demonstrated that empagliflozin (EMPA) pretreatment could also reduce DOX-induced cardiomyocyte toxicity, improve cell survival rate by alleviating ERS, and simultaneously inhibit oxidative stress and inflammatory responses (181).

Shengmai Injection (SMI) is an intravenous preparation of traditional Chinese medicine. It has been long used in China for treating heart failure of various etiologies (182–184) and exhibits the effect of inhibiting cardiomyocyte apoptosis (185). Further experimental studies demonstrated that SMI could significantly downregulate the expression of GRP78 and caspase-12, reduce ERS and ERS-related apoptosis, and ultimately improve cardiac function in DIC rats (186). Indole derivatives are substances widely present in natural plants and their metabolites. They possess multiple biological effects, including antioxidant, mitochondrial protective, anti-inflammatory, and ERS-alleviating properties (187, 188), and can counteract myocardial injury in DIC (189).

## 5 Discussion

The ER is a core organelle involved in critical cellular processes, including protein synthesis, folding, modification, transport, regulation of intracellular calcium levels, and lipid biosynthesis. Under normal conditions, the ER maintains proper protein folding and intracellular homeostasis. However, when cells are exposed to stresses such as hypoxia, nutrient deprivation, or infection, ERS occurs, disrupting its normal functions. This disruption triggers a cellular response known as the UPR, which is aimed at restoring ER homeostasis. The UPR involves three key signaling pathways—PERK, IRE1, and ATF6—to alleviate the accumulation of unfolded proteins caused by ERS. While these pathways help restore intracellular homeostasis, their prolonged activation may lead to cell death if the stress remains unresolved.

DCM is defined by progressive ventricular dilation and systolic dysfunction, contributing significantly to global heart failure mortality and remaining a leading indication for cardiac transplantation. ERS-induced apoptosis contributes to DCM pathogenesis. Pathogenic variants in genes such as *BAG5* and *LMNA*, alongside epigenetic regulators (e.g., miR-16-5p), modulate ERS in cardiomyocytes, accelerating disease onset and progression. These factors disrupt critical intracellular processes—including protein folding, autophagy, and mitochondrial function—culminating in myocardial injury and heart failure.

DMCM is a common complication of diabetes, characterized by myocardial remodeling and dysfunction. Multiple factors, such as estrogen, ARRDC4, IL-33, G-CSF, and H₂S, influence DMCM by regulating ERS-related proteins, autophagy, and key signaling pathways (e.g., PI3K/AKT). Dysregulation of these pathways exacerbates ERS, leading to oxidative stress, inflammation, and fibrosis, which accelerate the progression of heart failure. Additionally, molecules such as PI3K, GCN2, and BRD7 regulate ERS in DMCM, providing novel therapeutic targets for intervention.

HCM is predominantly caused by sarcomeric pathogenic variants, leading to abnormal myocardial thickening, diastolic dysfunction, and heart failure. Animal studies demonstrate that seipin deficiency induces HCM associated with ERS, inflammation, and cardiomyocyte apoptosis. Impaired calcium handling and defective protein folding exacerbate myocardial hypertrophy. Furthermore, ERS-activated PERK/ATF4 signaling drives HCM progression, highlighting the complex interplay between pathogenic variants, ERS, and cellular stress in HCM pathogenesis.

ARVC is a progressive disease characterized by the replacement of myocardial tissue with fibrofatty and fibrous tissue, leading to ventricular arrhythmias and SCD. ERS plays a critical role in ARVC, as evidenced by abnormal autophagy in cardiomyocytes, misfolding of the pathogenic gene DSG2, and alterations in ERS-related markers. Hyperactivation of the ATF4/TGF-β1 signaling axis is associated with myocardial fibrosis in ARVC, representing a potential therapeutic target. Studies on the molecular mechanisms of ERS in ARVC have identified key signaling pathways and genetic factors contributing to disease progression, opening new avenues for targeted therapy.

Significant progress has been made in understanding the role of ERS in various cardiomyopathies, uncovering complex molecular networks that govern disease development. Key genetic and proteomic factors regulating ERS—including the PERK, IRE1, and ATF6 pathways—have been identified. These findings provide valuable insights into the molecular mechanisms driving disease progression and offer multiple targets for the prevention and treatment of cardiomyopathies.

However, despite these advances, clinical management remains challenging. While heart transplantation remains a key therapeutic approach for end-stage heart failure, there is an urgent need for novel therapies targeting underlying molecular mechanisms, particularly those related to ERS. Clinically, the treatment of ERS-related cardiomyopathies is still in its early stages. Current strategies focus on symptom management, prevention of disease progression, and improvement of cardiac function. Pharmacological interventions such as β-blockers, ACEIs, and aldosterone antagonists are commonly used to reduce cardiac workload and alleviate heart failure symptoms (190, 191). However, these therapeutic approaches do not directly target ERS-related mechanisms. Recent studies have suggested that chaperones facilitating protein folding may have therapeutic potential, exerting effects by alleviating ERS and improving cellular function. For instance, compounds such as tauroursodeoxycholic acid (TUDCA) (192) and 4-phenylbutyric acid (4-PBA) (82, 141, 193) have shown promise in preclinical studies, and clinical trials are currently underway to evaluate their efficacy and safety in treating ERS-related cardiomyopathies.

Gene therapy and molecular interventions targeting UPR components (e.g., PERK, IRE1, or ATF6) hold great therapeutic potential. Modulating these pathways may enhance cardiomyocyte responses to ERS, preventing cell death and improving cardiac function. Additionally, emerging therapies targeting inflammation and fibrosis—such as anti-TGF-β1 antibodies—are being explored as adjunctive approaches to address the downstream effects of ERS in cardiomyopathies.

In conclusion, ERS is a key factor in the pathophysiological processes of various cardiomyopathies, contributing to myocardial dysfunction, apoptosis, and disease progression. While significant progress has been made in understanding the molecular mechanisms of ERS—particularly in the context of DCM, DMCM, HCM, and ARVC—translating these insights into effective clinical therapies remains challenging. The identification of key signaling pathways (e.g., PERK, IRE1, ATF6) has opened new avenues for targeted therapies aimed at alleviating ERS and improving cardiac function. However, current therapeutic options remain limited, with heart transplantation often being the last resort for end-stage heart failure. Thus, there is an urgent need for novel approaches (e.g., chaperones, gene therapy, and UPR-modulating molecular interventions) to address the underlying molecular mechanisms of ERS. Future research should focus on developing and refining these therapeutic strategies, with an emphasis on personalized medicine based on individual genetic profiles. Clinical trials investigating the efficacy of these innovative therapies—especially in reducing ERS-related damage and improving patient outcomes—should be prioritized. By advancing our understanding of ERS and implementing targeted therapies, we can significantly improve outcomes for patients with cardiomyopathies, enhance their survival rates and quality of life, and ultimately reduce the global burden of cardiovascular diseases.
